# Supplementary material for: Accounting for corner flow unifies the understanding of droplet formation in microfluidic channels
Source: Nat Commun. 2019 Jun 7;10:2528. doi: 10.1038/s41467-019-10505-5 (PMC6555794; doi:10.1038/s41467-019-10505-5)
Supplement: Supplementary file 1 — Supplementary Information [file 41467_2019_10505_MOESM1_ESM.pdf]

SUPPLEMENTARY INFORMATION

Accounting for corner flow unifies the understanding of droplet formation in microfluidic channels

PIOTR M. KORCZYK[1]*†, VOLKERT VAN STEIJN[2]*†, SLAWOMIR BLONSKI[1], DAMIAN ZAREMBA[1], DAVID A. BEATTIE[3] AND PIOTR GARSTECKI[4]*

1 Institute of Fundamental Technological Research, Polish Academy of Sciences, Pawinskiego 5B, 02-106 Warsaw, Poland

2 Delft University of Technology, Faculty of Applied Sciences, Dept. Chemical Engineering, van der Maasweg 9, 2629 HZ, Delft, The Netherlands

3 Future Industries Institute, University of South Australia, Mawson Lakes Campus, Mawson Lakes, SA 5095, Australia

4 Institute of Physical Chemistry, Polish Academy of Sciences, Kasprzaka 44/52, 01-224 Warsaw, Poland

* Corresponding authors:
Piotr M. Korczyk: piotr.korczyk@ippt.pan.pl
Volkert van Steijn: v.vansteijn@tudelft.nl
Piotr Garstecki: garst@ichf.edu.pl

† These authors contributed equally to this work.

Supplementary Note 1: Comparison of circular and square channels

Additional experiments were performed to test the hypothesis that the Ca-dependence of the droplet length at low Ca observed in channels with square cross section only exists in channels that allow the formation of gutters. For this purpose, a T-junction with channels of circular cross section was fabricated by the use of micro-milling. Two symmetrical halves of the device were fabricated by the use of a ball-end milling bit. Bonding the top and bottom halves, circular channels were obtained with a diameter of Dia = 400 μm.

Supplementary Figure 1 shows the comparison of droplets produced in T-junctions with circular and square cross section. As expected, the Ca-dependence is very weak in circular channels in comparison to that in square channels where the length of droplets explodes as Ca → 0. This fundamental difference clearly demonstrates the significance of gutters in droplet formation at low Ca.

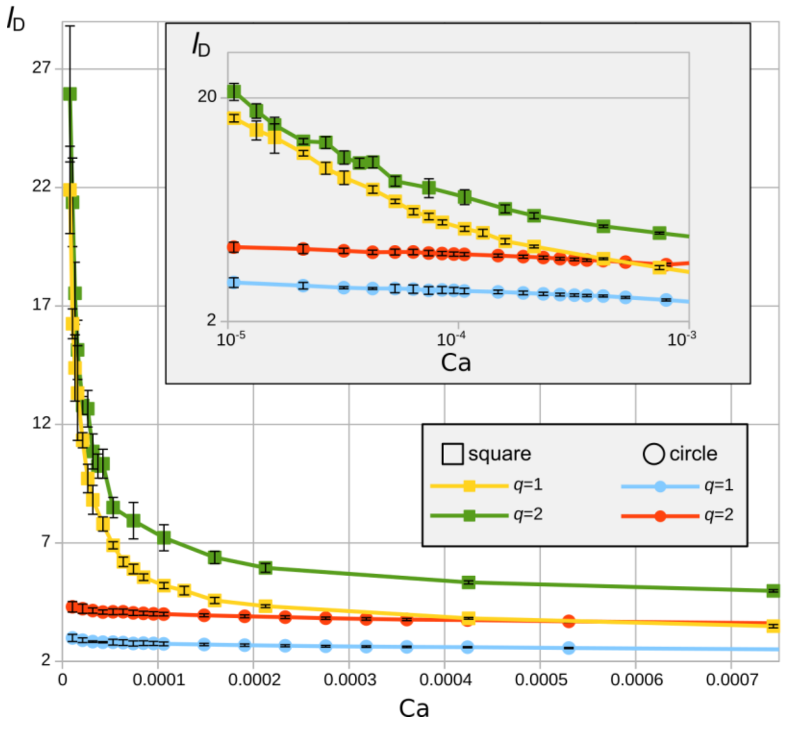

Supplementary Figure 1 Measurements of the length of droplets as a function of Ca for two ratios of flow rates q for T-junctions of square (W = H = 360 μm) and circular (Dia = 400 μm) cross section. The error bars represent the standard deviations of the measurements.

Supplementary Note 2: Full solution for Q_N

The full solution of Eq. 2 (from the main text) and the equivalent equation $\frac{\gamma V_\mathrm{N}^*}{C} = R_\mathrm{G}(t) \cdot (Q_\mathrm{C} - dV_\mathrm{N}^*/dt)/4$, with $Q_\mathrm{N} = \frac{dV_\mathrm{N}^*}{dt}$ and $R_\mathrm{G} = \frac{\alpha_\mathrm{G}\mu_\mathrm{C}}{A_\mathrm{G}^2}(L_0 + t \cdot Q_\mathrm{D}/WH)$, is:

$$Q_\mathrm{N}(t) = \frac{q\mathrm{Ca}}{q\mathrm{Ca}+\beta} Q_\mathrm{C} \cdot \left(1 - \left[\frac{t}{\tau_\mathrm{D} l_0} + 1\right]^{-\left(1+\frac{\beta}{q\mathrm{Ca}}\right)}\right) \qquad \text{Eq. (1)}$$

where $\beta = \frac{4}{\alpha_\mathrm{G}}\frac{A_\mathrm{G}^2}{C}$ and $l_0 = L_0/W$, with L_0 the initial length of the droplet at the start of the necking regime, and where $\tau_\mathrm{D} = \frac{W^2 H}{Q_\mathrm{D}} = W\frac{WH}{qQ_\mathrm{C}}$ is the time needed to increase the length of a droplet by W.

Two characteristic times appear in Supplementary Equation 1, which is easily seen when, for example, expanding the last term using a Taylor series for short *t*: one time scale corresponds to the fixed part of the resistance, $\frac{\alpha_\mathrm{G}\mu_\mathrm{C} C L_0}{4\gamma A_\mathrm{G}^2}\left(= \frac{q\mathrm{Ca}}{\beta}\tau_\mathrm{D} l_0\right)$, the second to the time-varying part, $\frac{L_0}{\frac{Q_\mathrm{D}}{WH}}(= \tau_\mathrm{D} l_0)$. The first can be seen as a classical RC time, while the second arises from the rate at which the DP is supplied. The first one is negligible compared to the second one in the leaking regime (with $\frac{q\mathrm{Ca}}{\beta} \ll 1$), while both are of the same order in the squeezing regime (where $\frac{q\mathrm{Ca}}{\beta}$ is of order 1). Importantly, for the conditions studied here, the influence of both time scales is negligible and $Q_\mathrm{N}(t)$ reaches a fixed value after a short initial transient. Before demonstrating this, we comment on the physical interpretation of the RC time. In classical RC circuits, the RC time corresponds to the time that is required for the relaxation of the system after a change in the potential applied over the circuit. Here, it can be interpreted as the relaxation of the interfacial surface to the equilibrium shape after a change in applied pressure. In the leaking regime, where the ratio between the two times scales, $\frac{q\mathrm{Ca}}{\beta}$, is small, the shape of

the interface almost instantaneously adapts to the rise in pressure due to the growth of the droplet. Hence, neck's shapes are close to the equilibrium shape in the leaking regime (see Fig. 4a in the main article).

The weak time-dependence of $Q_{\mathrm{N}}(t)$ is easily seen from an analysis of the time-dependent term $\left[\frac{t}{\tau_{\mathrm{D}} l_0}+1\right]^{-\left(1+\frac{\beta}{q\mathrm{Ca}}\right)}$ in Supplementary Equation 1. This term ensures the acceleration from $Q_{\mathrm{N}}=0$ at the start of the necking stage to $Q_{\mathrm{N}}(t)=\frac{q\mathrm{Ca}}{q\mathrm{Ca}+\beta}Q_{\mathrm{C}}$. Analysis of this term reveals that it decays very fast in comparison to the generation rate of long droplets, especially for $q\mathrm{Ca}<\beta$. For example, the time required for this term to affect $Q_{\mathrm{N}}(t)$ by less than 10% follows from $\left[\frac{t}{\tau_{\mathrm{D}} l_0}+1\right]^{-\left(1+\frac{\beta}{q\mathrm{Ca}}\right)}<0.1$. Putting in $\frac{\beta}{q\mathrm{Ca}}=1$, we obtain: $\frac{t}{\tau_{\mathrm{D}} l_0}>2.16$. Long droplets formed at low Ca require a much longer time than this ($\frac{L_{\mathrm{D}}}{W}\sim\frac{t}{\tau_{\mathrm{D}}}$). In fact, we used $L_{\mathrm{D}}/W>2.5$ as the minimum required length for the squeezing mechanism to be operative, based on the observation that shorter droplets do not fully occupy the junction before pinching off (in Fig. 1). For all droplets formed in the leaking regime ($q\mathrm{Ca}<\beta$), the initial transient hence is short compared to the total formation time such that droplets propagate at a constant velocity during most of the necking stage. This theoretical analysis justifies why the time-dependent term in Supplementary Equation 1 can be neglected. This justification was verified with additional experiments, described next in Supplementary Note 3.

Supplementary Note 3: Experimental verification of time-independent Q_N

To experimentally verify the outcome of the above analysis, we estimated the instantaneous flow rate to the neck, Q_N, by measuring the speed of the tip of forming droplets[1,2] using a high-speed camera. We here use the notion that the position of the tip, X_F, propagates at a rate equal to the sum of the flow rates of the neck and the DP

$$\frac{dX_\mathrm{F}}{dt}WH = Q_\mathrm{N} + Q_\mathrm{D} \qquad \text{Eq. (2)}$$

or in dimensionless quantities

$$\frac{dx_\mathrm{F}}{dt^*} = Q_\mathrm{N}/Q_\mathrm{C} + q \qquad \text{Eq. (3)}$$

where we normalized the position using $x_\mathrm{F} = X_\mathrm{F}/W$ and the time using $t^* = tQ_\mathrm{C}/HW^2$. We measured X_F (see the inset in Supplementary Figure 2a) for different Ca and fixed $q = 1$. A plot of x_F versus t^* shows that the tip position increases at a constant rate during most of the necking stage, i.e. $\frac{dx_\mathrm{F}}{dt^*}$ is constant in time for each curve (Supplementary Figure 2a). As evident from Supplementary Equation 3, this implies that $Q_\mathrm{N}/Q_\mathrm{C}$ is constant in time during the necking stage (and so are Q_B and $\eta = Q_\mathrm{B}/Q_\mathrm{N}$).

The notion that the slope of the curves in Supplementary Figure 2a is a measure for the gutter flow rate is used to test the consistency of the theoretical relation $\bar{\eta} = \frac{\beta}{q\mathrm{Ca}}$ together with the value $\beta = 7.4 \cdot 10^{-5} \pm 0.3 \cdot 10^{-5}$ reported in the main article, which was obtained from measurements of the final droplet length. Rewriting Supplementary Equation 3 in terms of $\eta = \frac{Q_\mathrm{B}}{Q_\mathrm{N}}$ using $\frac{Q_\mathrm{N}}{Q_\mathrm{C}} = \frac{Q_\mathrm{N}}{Q_\mathrm{N}+Q_\mathrm{B}} = \frac{1}{1+\eta}$ gives

$$\eta = \left(\frac{dx_\mathrm{F}}{dt^*} - q\right)^{-1} - 1 \qquad \text{Eq. (4)}$$

such that the (time-averaged) leaking strength $\bar{\eta}$ can be obtained from the (time-averaged) slopes $\overline{\frac{dx_\mathrm{F}}{dt^*}}$ of the curves in Supplementary Figure 2a according to

$$\overline{\eta} = \left(\frac{\overline{dx_{\mathrm{F}}}}{dt^*} - q\right)^{-1} - 1 \qquad \text{Eq. (5)}$$

The thus obtained values of $\overline{\eta}$ confirm the earlier reported theoretical relation $\overline{\eta} = \frac{\beta}{q\mathrm{Ca}}$ and the reported value for β as shown in Supplementary Figure 2b.

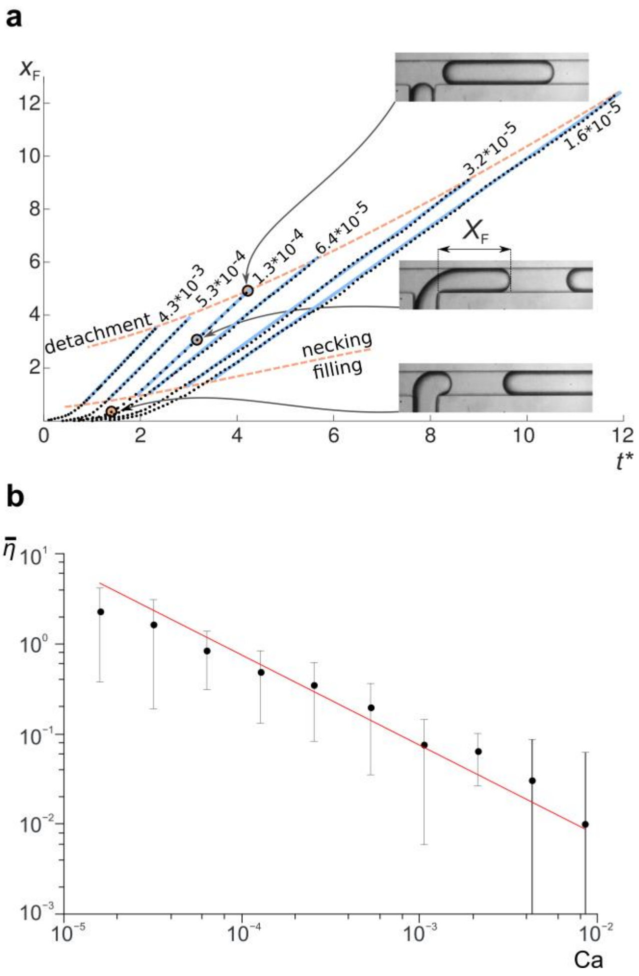

Supplementary Figure 2 Experimental analysis of the instantaneous tip speed and the corresponding time-averaged strength of leaking $\overline{\eta}$. (a) Dotted lines – normalized measurements of the position of the tip $x_{\mathrm{F}} = X_{\mathrm{F}}/W$ as a function of the normalised time: $t^* = tQ_{\mathrm{C}}/HW^2$. $t^* = 0$ is the start of the formation of a droplet. Different measurement sets correspond to different values of Ca from $1.6 \cdot 10^{-5}$ to $4.3 \cdot 10^{-3}$ as described in the plot. During the necking stage (highlighted by straight blue lines), x_{F} increases approximately linearly with time. (b) Time-averaged $\overline{\eta}$ as a function of Ca. $\overline{\eta}$ was calculated using Supplementary Equation 5, where $\overline{dx_{\mathrm{F}}/dt^*}$ was determined from the curves in (a) using a linear fit of the necking stage. Red line: $\overline{\eta} = \frac{\beta}{q\mathrm{Ca}}$ (where $q = 1$) with $\beta = 7.4 \cdot 10^{-5}$. This plot shows the consistency of these measurements with the analysis presented in the main text. All measurements made for $q = 1$ for the hexadecane-FC-40 fluid system in a T-junction with $W = H = 360$ μm.

Supplementary Note 4: Leaking regime for different working fluids

All experiments in the main article and the ones in the Supplementary Information up to here were performed with FC-40 (viscosity: 4.1 mPa·s) as the DP and hexadecane (viscosity: 3.6 mPa·s) as the CP, the interfacial tension being 7.3 $mN \cdot m^{-1}$. In order to show that the leaking regime described in the paper is not specific for this combination of liquids we performed additional experiments with different liquid combinations (see Supplementary Tables 1 – 3). Care was taken to choose these working fluids such that the CP wets the walls of the polycarbonate microchannels. While the combination of liquids used in the main article is surfactant free, the surfactant Span 80 (Sigma-Aldrich) was added in a 1%wt concentration for the other presented combinations to avoid wetting by the DP. The universality of the observed behaviour is shown in Supplementary Figure 3 for the different fluid systems.

Supplementary Table 1: Viscosities of liquids used in the experiments (at 22°C).

Symbol	Description	Viscosity [mPa·s]
CP1	hexadecane	3.6
CP2	~50 wt. % of hexadecane in n-hexane	0.93
CP3	~10% wt. % of hexadecane in n-hexane	0.38
DP1	~60 wt. % of glycerine in water	10.3
DP2	~40 wt. % of glycerine in water	3.7
DP3	water	0.95

Supplementary Table 2: Ratio of viscosities DP to CP (at 22°C).

	DP1	DP2	DP3
CP1	2.9	1.0	2.6
CP2	11.1	4.0	1.0
CP3	27.1	9.7	2.5

Supplementary Table 3: Interfacial tension (in $mN \cdot m^{-1}$) of the different combinations of liquids (at 22°C).

	DP1	DP2	DP3
CP1	3.6	4.1	4.8
CP2	4.4	4.6	5.3
CP3	4.8	4.9	5.4

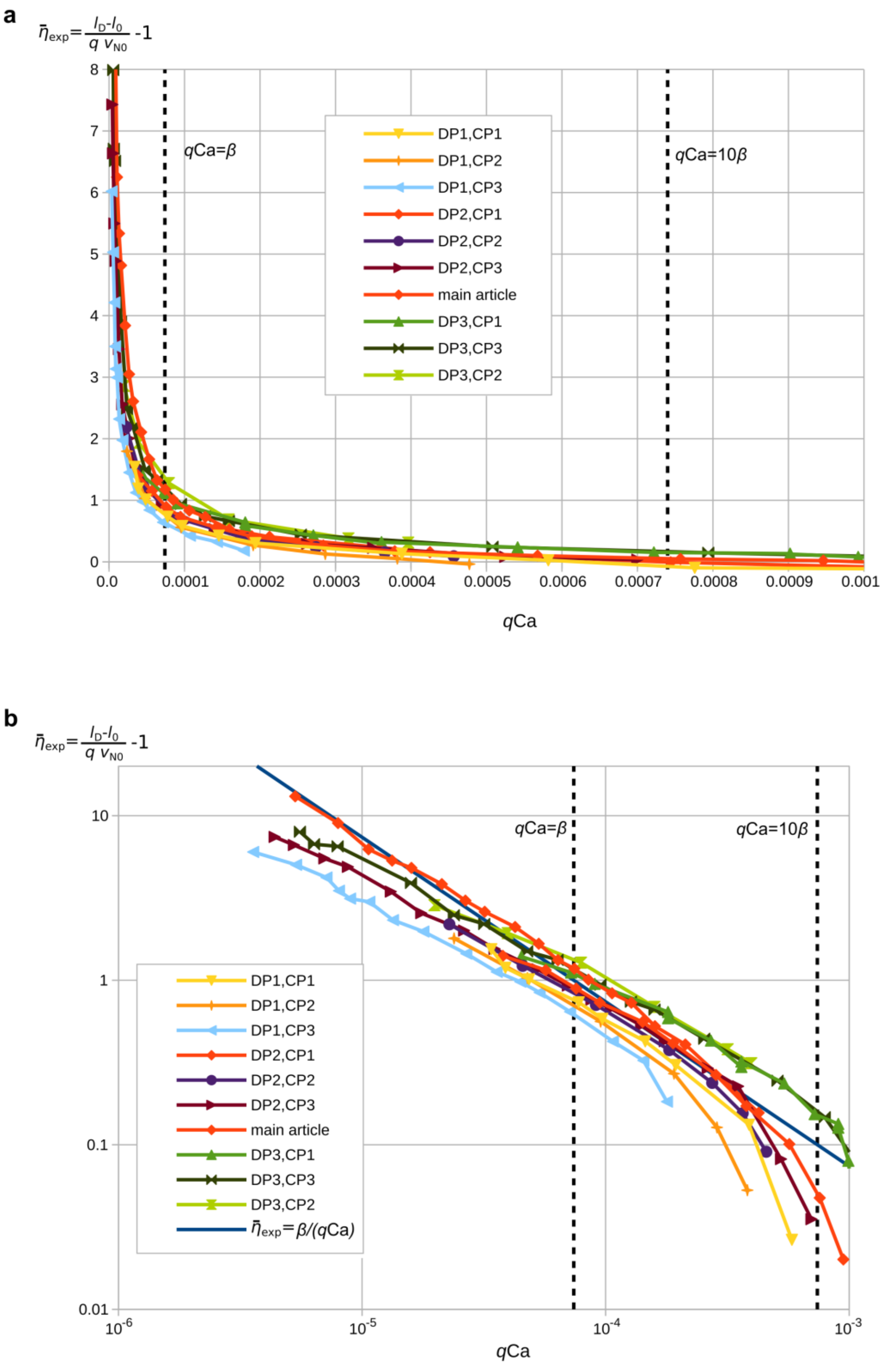

Supplementary Figure 3: The leaking regime and the L-S transition for different liquid properties (see legends and Supplementary Tables 1-3 for references): (a) linear plot, (b) log-log plot including the theoretical prediction $\bar{\eta} = \frac{\beta}{q\mathrm{Ca}}$. For all model lines, the same values were used as obtained in Fig. 3 ($l_0 = 1.46 \pm 0.14$, $v_{\mathrm{N0}} = 2.04 \pm 0.11$, and $\beta = 7.4 \cdot 10^{-5} \pm 0.3 \cdot 10^{-5}$).

Supplementary Note 5: Leaking regime for square channels of different widths

The equation for the time-averaged leaking strength ($\overline{\eta} = \frac{\beta}{q\mathrm{Ca}}$) provided in the main article is non-dimensional, and hence should not depend on the used channel width. We confirm this with additional experiments using a square channel of a different width (200 µm). Supplementary Figure 4 presents a comparison for $W = H = 200$ µm and $W = H = 360$ µm, showing that the two data sets indeed collapse on top of each other.

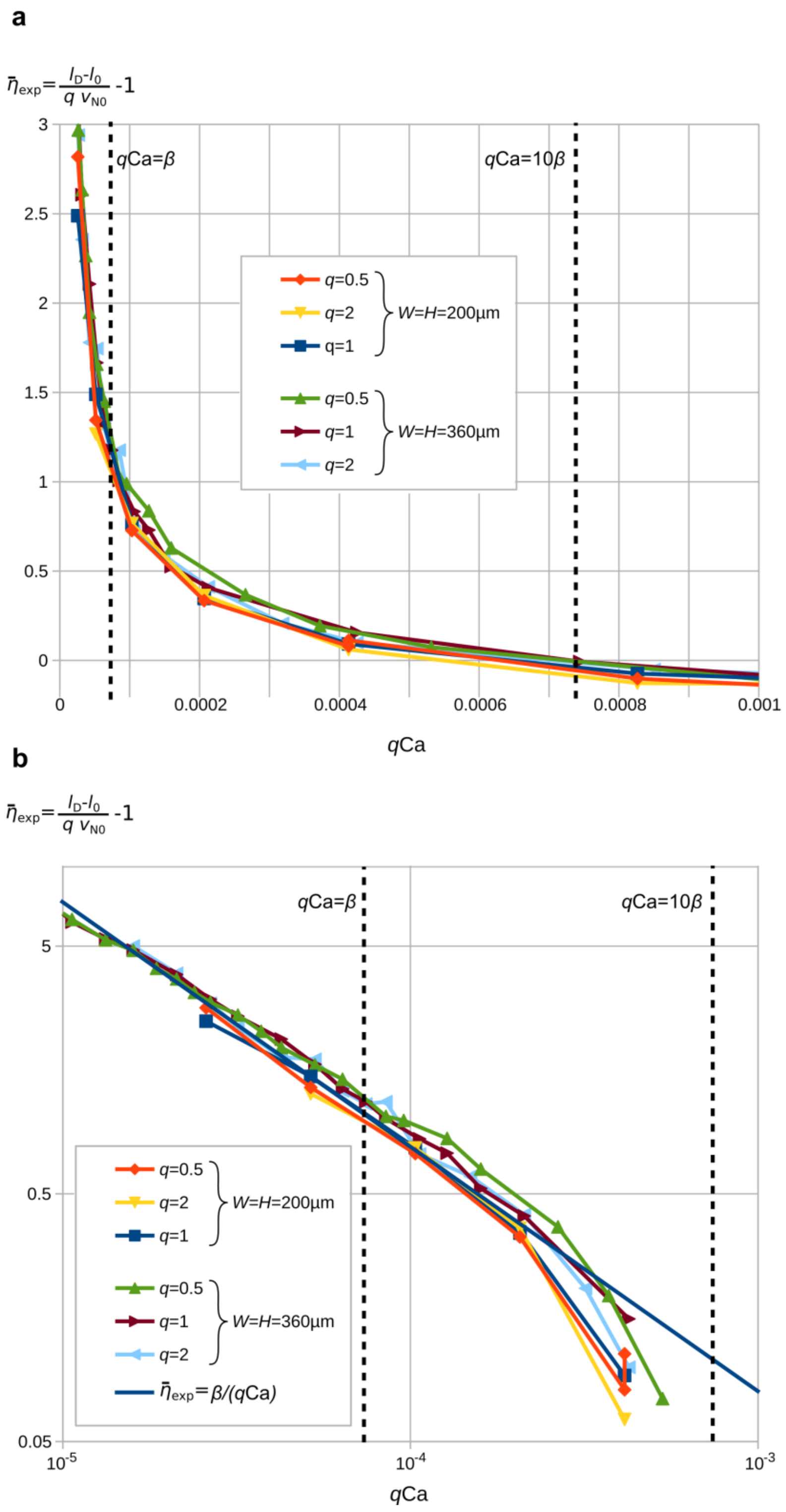

Supplementary Figure 4: Comparison of measurements for the leaking regime for two square channels of different widths for the hexadecane-FC-40 fluid system: (a) linear plot, (b) log-log plot including the theoretical prediction $\bar{\eta} = \frac{\beta}{q\mathrm{Ca}}$. For all model lines, the same values were used as obtained in Fig. 3 ($l_0 = 1.46 \pm 0.14$, $v_{\mathrm{N0}} = 2.04 \pm 0.11$, and $\beta = 7.4 \cdot 10^{-5} \pm 0.3 \cdot 10^{-5}$).

Supplementary Note 6: Leaking regime for different channel aspect ratios

The leaking mechanism is a feature of the formation of droplets in channels with non-circular cross section. We hence expect this mechanism to also feature in rectangular channels with different aspect ratios. As the aspect ratio imposes the curvature of the interfaces during droplet formation as well as the size of the gutters, the quantitative dependence of droplet size on Ca and q is expected to depend on the aspect ratio. Experiments performed in channels with aspect ratios $H/W = 0.5$ and $H/W = 1$ confirm that the general features are the same, while the quantitative features depend on aspect ratio, see Supplementary Figure 5 and Supplementary Table 4.

A closer look at the leaking regime shows a lower slope in the log-log plot for the channel with $H/W = 0.5$, suggesting that some details of the mechanism of leaking (such as the linear relation between the curvature difference and the volume collected behind the forming droplet) depend on the aspect ratio. A more extensive analysis of the effect of channel aspect ratio and cross-sectional shape is beyond the scope of the present paper and part of future work.

Supplementary Table 4: Comparison of fitting parameters for rectangular channels with different aspect ratio.

Aspect ratio H/W	l_0	v_{N0}	β
1	1.46 ± 0.14	2.04 ± 0.11	$7.4 \cdot 10^{-5} \pm 0.3 \cdot 10^{-5}$
1/2	2.27 ± 0.24	2.54 ± 0.37	$2.1 \cdot 10^{-5} \pm 0.1 \cdot 10^{-5}$

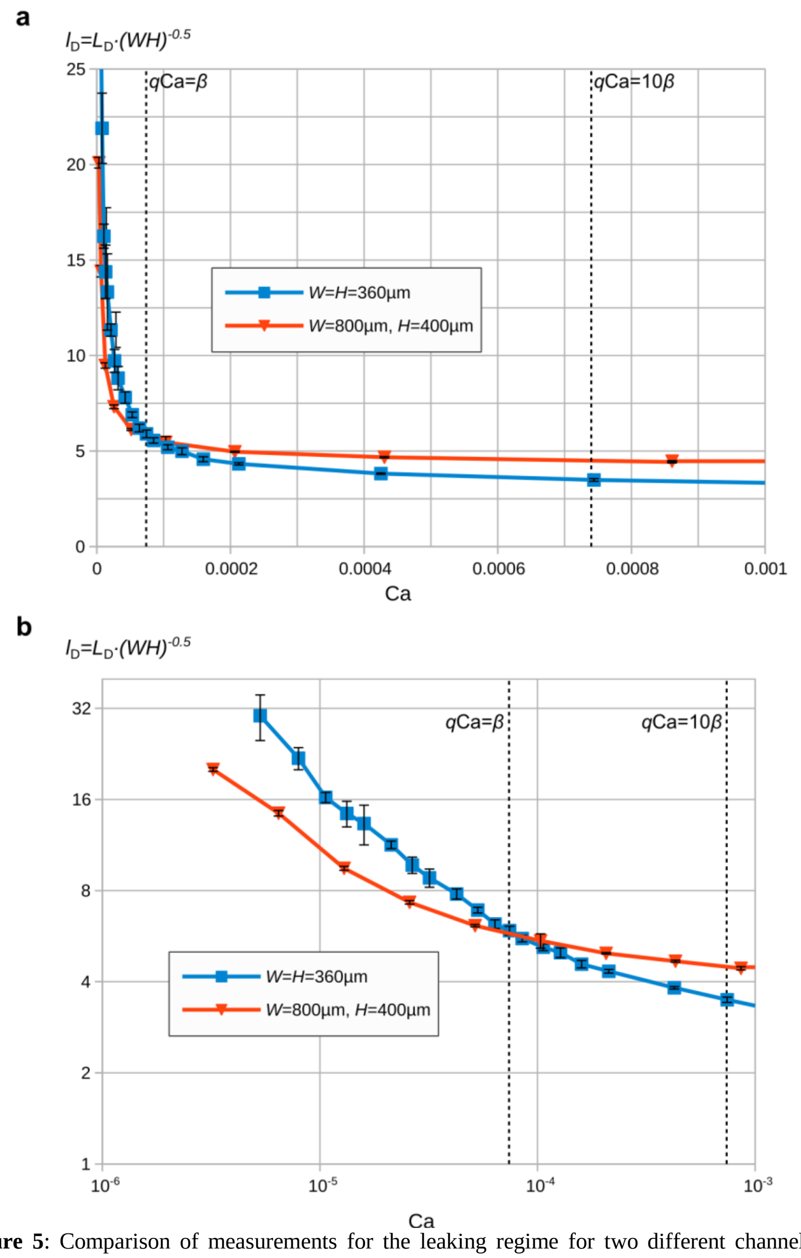

Supplementary Figure 5: Comparison of measurements for the leaking regime for two different channel aspect ratios for the hexadecane-FC-40 fluid system and $q = 1$: (a) linear plot, (b) log-log plot.

Supplementary Note 7: How droplet length depends on $q = Q_D/Q_C$ at fixed Q_C or Q_D

In Fig. 1b/c of the main article we showed experimental series for the length of droplets in which we varied Ca for fixed ratios of flow rates $q = Q_D/Q_C$. In order to also systematically test the functional dependence of the droplet length on q we performed additional series of experiments varying q for fixed values of Q_D or Q_C. Note that fixing Q_C fixes Ca, while fixing Q_D fixes qCa.

Let us consider again the model equation for the length of a droplet:

$$l_D = l_0 + q v_{N0}(1 + \frac{\beta}{q\mathrm{Ca}}) \qquad \text{Eq. (6)}$$

where $\mathrm{Ca} = \mu_C U/\gamma$ and $U = Q_C/HW$. Hence $\mathrm{Ca} = \varphi Q_C$, with $\varphi = \frac{\mu_C}{\gamma W H}$ $(= 1.06 \cdot 10^{-6}\ \mu\mathrm{l} \cdot \mathrm{h}^{-1}$ for the experiments with FC-40 as the DP and hexadecane as the CP).

Supplementary Equation 6 can be rewritten in two versions:

- The first variant for varying Q_D at fixed Q_C, which also fixes Ca, such that the droplet length linearly depends on q, according to

  $$l_D = l_0 + q v_{N0} + \frac{\beta v_{N0}}{\varphi Q_C} \qquad \text{Eq. (7)}$$

  Curves of l_D versus q for different values of Q_C are hence expected to have the same slope (v_{N0}), but different offsets ($l_0 + \frac{\beta v_{N0}}{\varphi Q_C}$). This is indeed the case, see Supplementary Figure 6a.

- The second variant for varying Q_C at fixed Q_D, which also fixes qCa, such that the droplet length still linearly depends on q, now according to

  $$l_D = l_0 + q v_{N0} \left(1 + \frac{\beta}{\varphi Q_D}\right) \qquad \text{Eq. (8)}$$

  Curves of l_D versus q for different values of Q_D should hence have the same offset (l_0) but different slopes $v_{N0}\left(1 + \frac{\beta}{\varphi Q_D}\right)$, which is indeed the case, see Supplementary Figure 6b.

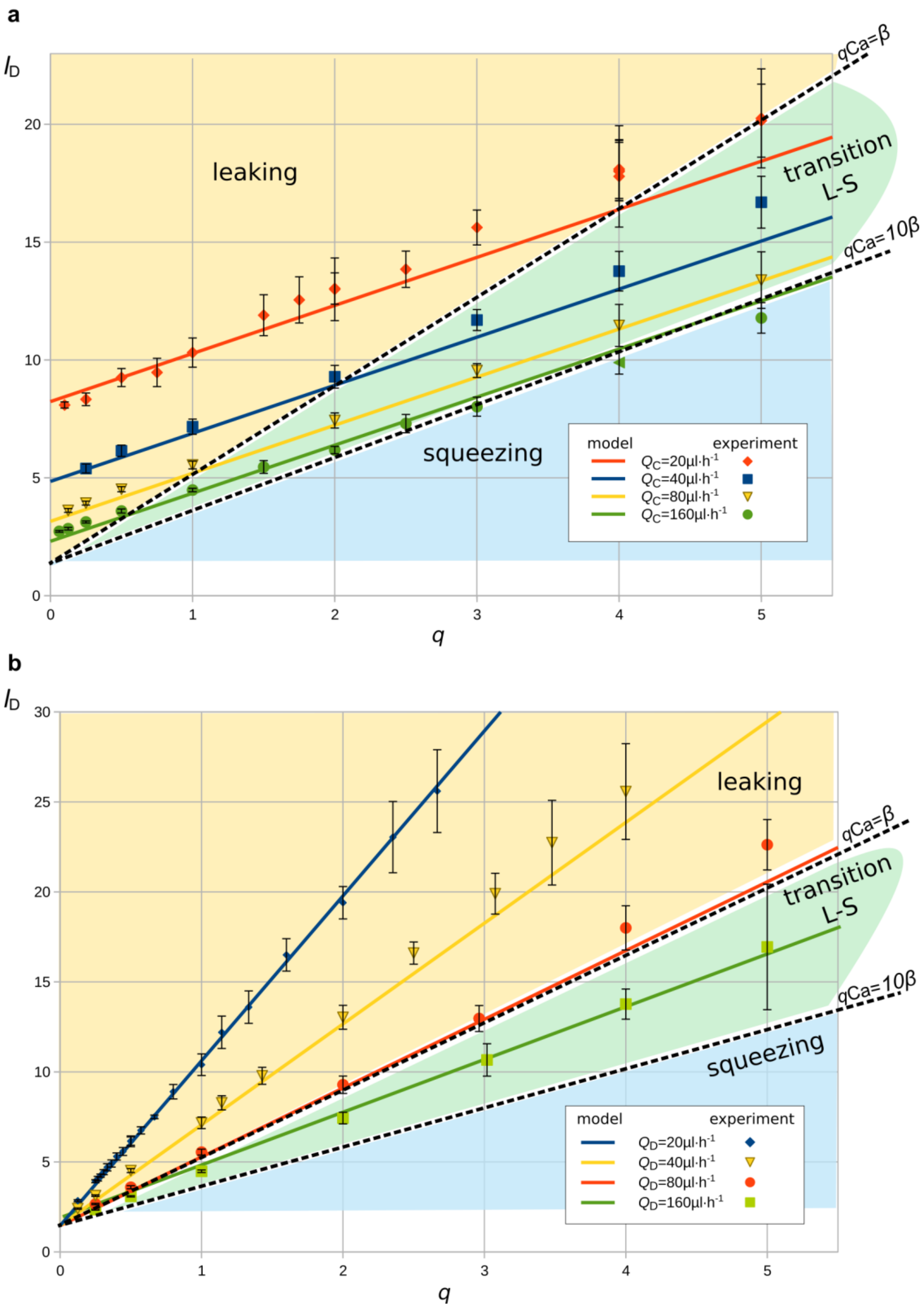

Supplementary Figure 6 Normalised length of droplets as a function of $q = Q_D/Q_C$ for the hexadecane-FC-40 fluid system in a T-junction with $W = H = 360$ µm. (a) for four measurement series with varying Q_D at fixed Q_C; model: Supplementary Equation 7. (b) for four measurement series with varying Q_C at fixed Q_D; model: Supplementary Equation 8. For all model lines, the same values were used as obtained in Fig. 3 ($l_0 = 1.46 \pm 0.14$, $v_{N0} = 2.04 \pm 0.11$, and $\beta = 7.4 \cdot 10^{-5} \pm 0.3 \cdot 10^{-5}$).

Supplementary Note 8: Stability of feeding system

Our previous work[3] showed that results of microfluidic experiments may be affected by oscillations in flow due to non-continuous rotation of the screw of the syringe pumps. In all experiments presented in this work we used precise syringe pumps (Cetoni, Low Pressure Syringe Pump neMESYS 290N) designed to be used in microfluidics. In order to verify that our measurements in the leaking regime are not influenced by pump oscillations, we carried out additional experiments with syringes of a different diameter since oscillations are known to be depended on syringe size. The data presented in Supplementary Figure 7 show that the results for different syringes are almost identical, confirming that there are no significant temporal oscillations in our feeding system.

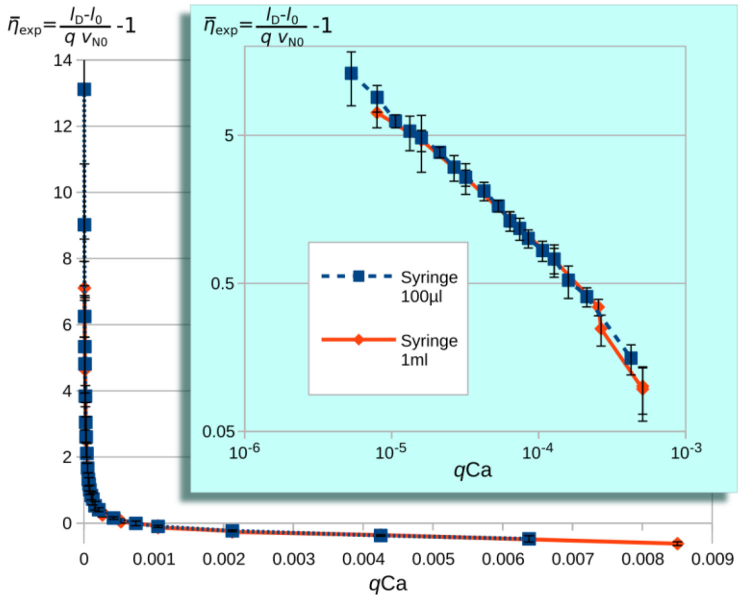

Supplementary Figure 7: Measurements of the time-averaged leaking strength in the leaking regime for two different syringe sizes for the hexadecane-FC-40 fluid system in a T-junction with $W = H = 360$ µm and for the ratio of flows $q = 1$. Inset –log-log plot of the same data as in the main plot. Dashed line – theoretical curve for the same model parameters as in the main article ($l_0 = 1.46 \pm 0.14$, $v_{\mathrm{N0}} = 2.04 \pm 0.11$, and $\beta = 7.4 \cdot 10^{-5} \pm 0.3 \cdot 10^{-5}$).

Supplementary References

1. Shui, L., van den Berg, A. & Eijkel, J. C. T. Capillary instability, squeezing, and shearing in head-on microfluidic devices. *J. Appl. Phys.* **106,** 124305 (2009).
2. Chen, X., Glawdel, T., Cui, N. & Ren, C. L. Model of droplet generation in flow focusing generators operating in the squeezing regime. *Microfluid. Nanofluidics* **18,** 1341–1353 (2014).
3. Korczyk, P. M., Cybulski, O., Makulska, S. & Garstecki, P. Effects of unsteadiness of the rates of flow on the dynamics of formation of droplets in microfluidic systems. *Lab Chip* **11,** 173–175 (2011).
